# Colonoscopy with Polypectomy in Patients Taking Clopidogrel

**DOI:** 10.4021/gr2009.08.1308

**Published:** 2009-07-20

**Authors:** Shai Friedland, Cynthia W Leung, Roy M Soetikno

**Affiliations:** a Department of Gastroenterology, VA Palo Alto Health Care System and Stanford University, Palo Alto, California, USA

**Keywords:** Colonoscopy, Colon cancer, Colon polyp, Polypectomy, Antiplatelet drug

## Abstract

**Background and Aims:**

To investigate the bleeding risk of colonoscopy with polypectomy in patients taking clopidogrel.

**Methods:**

Retrospective review of patients undergoing colonoscopy with polypectomy without interruption of clopidogrel. Patients with lesions larger than 1cm were generally rescheduled for polypectomy off clopidogrel. Most of the polyps were removed using cold snare technique. Endoscopic clips were routinely applied prophylactically.

**Results:**

A total of 125 polypectomies were performed in 60 patients. The average polyp size was 5.4 ± 2.1 mm. One patient (1.7%, CI 0.3-8.9%) developed post-polypectomy bleeding that resolved without treatment. Three patients (5%, CI 1.7-14%) had immediate bleeding during the procedure and all resolved with prompt clip application.

**Conclusions:**

Polypectomy of lesions up to 1cm in size can be performed without interruption of clopidogrel.

## Introduction

Clopidogrel is a thienopyridine derivative that irreversibly inhibits platelet activation and aggregation [1]. It is commonly used to reduce thrombotic events, particularly in patients who have undergone coronary stent placement [2, 3]. Temporary discontinuation of clopidogrel, particularly in patients who have undergone coronary stent placement within 3 - 6 months, is generally considered inadvisable unless there is a compelling indication [4]. When clopidogrel is stopped to perform surgery or endoscopy, a 7 - 10 day period is generally recommended to allow time for adequate replacement of the platelet pool [4]. However, there is a general lack of consensus in the literature as to the optimal strategy for balancing the risk of increased bleeding if clopidogrel is continued perioperatively and the risk of thrombotic events if it is interrupted [4, 5]. This ambiguity is also present in the 2005 American Society of Gastrointestinal Endoscopy guidelines for endoscopic procedures: for “high risk” procedures such as colonoscopic polypectomy, the guidelines state that “Whether to discontinue these agents has not been determined [6].” One of the fundamental difficulties with deciding how to manage clopidogrel in patients undergoing colonoscopy is that the risk of post-polypectomy bleeding in this setting is not known [6-8]. Our anecdotal experience has suggested that the bleeding risk is not exceptionally high when small polyps are resected, and based on this we have been routinely performing polypectomy of lesions up to 1 cm in size without interruption of clopidogrel [9].

## Materials and Methods

A retrospective chart review was conducted on patients undergoing colonoscopic polypectomy without interruption of clopidogrel between January 2007 and August 2008 at the VA Palo Alto Health Care System. The study was conducted with institutional review board approval. Our standard clinical practice, which was followed in all but 3 cases, was to perform polypectomy of lesions up to 1cm in size in these patients. Polyp size was estimated by comparison to a 10 mm snare or an opened biopsy forceps. Patients with larger lesions were rescheduled in order to perform polypectomy after interruption of clopidogrel for 1 week. In 3 cases, polyps larger than 1cm were removed, and these patients were also included in the series. In all patients, immediately following polypectomy, one or more endoscopic clips were placed prophylactically to close the polypectomy defect. Immediate bleeding was diagnosed when the endoscopist noted excessive (more than 20 ml) bleeding between the polypectomy and the placement of endoscopic clips. All patients were followed-up by telephone or in clinic at least 1 week after the procedure.

## Results

A total of 125 polypectomies were performed in 60 patients. Patient characteristics are described in Table 1. All of the patients were male, with an average age of 65. The majority of the colonoscopies, 78%, were performed on asymptomatic patients in our colorectal cancer screening/surveillance program, 63% of the patients were taking clopidogrel because of prior placement of coronary stents, 30% were taking the medication to prevent recurrent cerebrovascular events.

**Table 1:** Patient Characteristics

Number of Patients	60
Male (Female)	60 (0)
Average Age (Std Dev)	64.7 (8.1)
Indication for Colonoscopy (%)	
Prior polyp or cancer	24 (40%)
Screening	23 (38%)
Iron deficiency anemia	6 (10%)
Bleeding	3 (5%)
Other	4 (7%)
Indications for Clopidogrel (%)	
Coronary stent	38 (63%)
Stroke or transient ischemic attack	18 (30%)
Peripheral vascular disease	5 (8%)
Coronary disease without stent	3 (5%)
Concomitant Use of Other Medications (%)	
Aspirin	10 (17%)
Warfarin (held for 36 hours)	2 (3%)

The polyps that were removed ranged in size from 3 to 12 mm, with an average diameter of 5.4 mm (Table 2). Most of the polyps were removed either using cold snare technique [10] (snare without cautery) or by endoscopic mucosal resection [11] (snare with cautery following submucosal injection of saline). There were 3 episodes of immediate bleeding (5% of patients, CI 1.7-14%); all were successfully treated by endoclip application and did not require hospital admission or observation. There was 1 episode of delayed bleeding (1.7% of patients, CI 0.3-8.9%) that occurred 36 hours following resection of a 5 mm and a 6 mm adenoma by endoscopic mucosal resection. The patient had four grossly bloody bowel movements at home, but did not seek medical attention. The bleeding resolved without treatment. Photographs from the procedure were reviewed and no obvious irregularities in technique were observed. Both polypectomy sites appeared to have been clipped adequately at the time of the procedure.

**Table 2:** Polypectomy Characteristics

Number of Polyps	125
Polyps per patient (range)	2.1 (1-7)
Average Size (Std Dev)	5.4 mm (2.1 mm)
Size Range	3 – 12 mm
Polypectomy Technique	
Snare without cautery	91 (73%)
Snare with cautery after saline injection	27 (22%)
Snare without cautery after saline injection	3 (2%)
Biopsy forceps without cautery	3 (2%)
Snare with cautery after endoloop	1 (1%)
Histology	
Adenoma	98 (78%)
Non-neoplastic	14 (11%)
Specimen lost	13 (10%)

## Discussion

Clopidogrel is widely used for prevention of thrombotic events, particularly in patients who have undergone coronary stenting [12-14]. Optimal management of these patients requires knowledge of the bleeding risks associated with continuation of clopidogrel during colonoscopic polypectomy and the thrombotic risks that patients are exposed to if the medication is interrupted. While there has been significant attention paid to the potential risk of stopping clopidogrel in coronary stent patients, the bleeding risk associated with polypectomy has not been quantified systematically [15-17]. This retrospective series of 60 patients demonstrates that the risk of major bleeding is low. Only 1 patient (1.7%, CI 0.3-8.9%) had significant post-polypectomy bleeding, which was self limited and resolved without hospital admission. There were 3 episodes of immediate bleeding, all of which stopped with prompt clip application. This is rather subjective, as there is often some bleeding immediately after snaring of polyps when minimal or no cautery is utilized. However, the degree of immediate bleeding seemed excessive in these 3 cases and prompt clip application was necessary. It should also be noted that clips were placed prophylactically in all cases even when no bleeding was observed.

Limitations of this study include the single-center, retrospective design in a primarily male Veteran population. Technical aspects which may have contributed to the low post-polypectomy bleeding rate include the widespread use of cold snare technique (snare without cautery), and the use of submucosal injection when cautery was applied. It is plausible, although unproven, that these methods reduce the risk of wall injury and subsequent delayed bleeding [11]. Immediate clip application is also of unproven benefit, particularly since a randomized study in average bleeding-risk patients did not show a benefit in prevention of post-polypectomy bleeding [18]. However, anecdotally we commonly observed increased oozing following cold snare polypectomy in patients taking clopidogrel compared to average-risk patients, and in the absence of data on the bleeding risks we felt compelled to proceed with routine immediate clipping in all cases.

In conclusion, this series suggests that it is possible to perform colonoscopic polypectomy on lesions up to 1 cm in size without interruption of clopidogrel. Given that only a small minority of patients undergoing screening colonoscopy have lesions larger than 1 cm, it may be reasonable to consider performing screening examinations without stopping clopidogrel and rescheduling only those patients with larger lesions.
